# Void Content and Mechanical Properties of Carbon Fiber/Epoxy Composites with Different Stacking Sequences by Double-Vacuum-Bag Process

**DOI:** 10.3390/polym18161996

**Published:** 2026-08-16

**Authors:** Liangliang Ren, Yuze Kang, Yang Zhang

**Affiliations:** AVIC Composite Corporation Ltd., Beijing 101300, China

**Keywords:** carbon fiber/epoxy composites, laying modes, void, DVB process, mechanical properties

## Abstract

In the manufacturing of carbon fiber/epoxy composites, different stacking sequences have different effects on the void inside materials. In this paper, the double-vacuum-bag (DVB) process was utilized to fabricate laminates with different stacking sequences, including different angles, different thicknesses and plain weave prepregs, and the single-vacuum-bag (SVB) process was set as the control group. The void content of different laminates in the cross-section was counted by image analysis software, and material thickness, density, and fiber volume fraction were measured by experiments. Three-point bending and short-beam-shear tests were conducted to evaluate the material mechanical properties. The results show that the void contents of laminates prepared by the DVB process are all less than 1% with different stacking sequences, while the laminates manufactured by the SVB process contain a large number of voids inside. The density and fiber volume fraction of the DVB process are higher than those of the SVB process. In terms of mechanical properties, the flexural strength and interlayer-shear-strength (ILSS) of the DVB process are higher than those of the SVB process. The results of this paper expand the application of the DVB process and provide a reference for low-cost manufacturing of composite materials.

## 1. Introduction

Composite materials have been widely used in aerospace, automotive, shipbuilding, wind power and other industrial fields due to their excellent mechanical properties [1,2,3,4,5]. During fabrication, voids are unfilled areas in resin, and the factors that contribute to the formation of a void vary depending on the process [6,7,8]. In the manufacturing process of composite components based on prepregs, the volatiles generated during the curing cycle and the air retained during laying are the main reasons for void formation [9,10].

A void is an unfavorable factor in the material mechanical properties, and reducing void content is necessary to maintain material properties [11,12,13]. Li et al. found that the void content of the unidirectional prepregs increased from 0.7% to 5.4% as the curing pressure decreased from 0.6 MPa to 0.0 MPa, while the tensile strength decreased by 15.3% [14]. The ILSS of composite materials exhibits strong sensitivity to void content; for every 1% increase in void content, the ILSS typically decreases by 10% [15,16]. Through three-point bending tests, Hayashi et al. found that the flexural strength of carbon fiber-reinforced polypropylene cross-laminate decreases sharply when the void content is higher than 1% [17]. The autoclave process can compact materials with compressed gas, resulting in a low void content in composite components. However, this process has the disadvantages of high manufacturing cost and energy consumption, and it is necessary to develop out-of-autoclave (OoA) processes [18,19,20].

Based on the requirements of mechanical properties, different stacking sequences and prepreg types are applied in the manufacturing of composites, which can affect the size and distribution of voids [21]. Mehdikhani et al. used X-ray computed tomography technology to obtain 3D scanning images of carbon fiber/epoxy laminates with different stacking sequences; they found that the stacking sequences affected the size and shape of voids, and the voids were mainly concentrated at the central layer. The thicker the laminate, the more internal voids there were [22]. In addition, the voids inside the fiber bundles of woven composite materials were narrow and elongated and distributed along the fiber direction. Due to the accumulation of a large amount of resin at the intersection of fiber bundles, voids can be concentrated in this area [23,24,25].

The DVB process is a new OoA technology, where an outer vacuum bag is installed above the vacuum bag on the basis of the SVB process, and a breathable support mold is placed between the two bags to prevent the outer bag from collapsing during vacuuming. The outer and inner bags are completely evacuated, and the inner bag is pulled up to help remove trapped air and volatile substances. In the final curing stage, the outer bag is connected to the atmosphere, and the inner bag is evacuated to the maximum extent to compact the prepregs [26]. Hou et al. compared the SVB process and DVB process, finding that the void content of the DVB process was lower, the lowest void content was 1.9%, and the mechanical properties of DVB samples were consistently higher, with ILSS increasing by 45%, flexural strength increasing by 35%, and tensile strength increasing by 8.5% [27,28]. During the co-curing of repair patches with thin-film adhesive and prepreg, Chong et al. used the DVB process to reduce the void content from 5.9% to 0.3%; the flexural strength and ILSS of 1D repair patches were improved by 7.3% and 14.8%, respectively, reaching the level of the autoclave process [29,30].

However, the above studies of the DVB process did not consider the influence of different stacking sequences and prepreg types on material void content. In this paper, the DVB and SVB processes are used to prepare composite materials with different stacking angles, different stacking thicknesses and plain weave prepregs. The void morphology was observed using an optical microscope, and the void content was measured and counted. Then the material density was measured using the buoyancy method, and the fiber volume fraction was measured by the combustion method. Finally, the three-point bending and short-beam-shear tests were carried out to compare the flexural strength and ILSS of the materials under different processes. The experimental results of this paper extend the application range of the DVB process and further prove that the process can manufacture high-quality composite structures at a low cost.

## 2. Materials and Methods

### 2.1. Materials

The unidirectional prepregs used in this paper are T700/GE10 (Weihai Guangwei Composite Materials Co., Ltd., Weihai, China), with a carbon fiber density of 1.8 g/cm^3^, an initial resin mass content of 33%, and a resin density of 1.32 g/cm^3^. The weaving form of prepregs in this paper is plain, compared with other forms of weaving; this form is more prone to voids because it has more interweaving points and more yarn buckling points. The plain weave prepregs are WP-3011/GE10 (Weihai Guangwei Composite Materials Co., Ltd., Weihai, China), with an initial resin mass content of 40% and a carbon fiber density of 1.78 g/cm^3^. The other auxiliary materials are provided by Easy Composite Ltd., (Beijing, China). Table 1 shows the materials stacking sequences. The stacking sequences for different stacking angles are [0]_16_, [90]_16_, and [0/+45/90/−45]_4S_, all of which are 16 layers. The stacking sequences with different laying thicknesses are [0/90]_3S_, [0/90]_5S_, [0/90]_7S_, which correspond to 12 layers, 20 layers, and 28 layers respectively. The stacking sequence of the plain composite material is [(0,90)]_10_, with 10 layers in total. The size of prepregs is 200 × 300 mm.

### 2.2. Manufacturing Method

**Figure 1:**
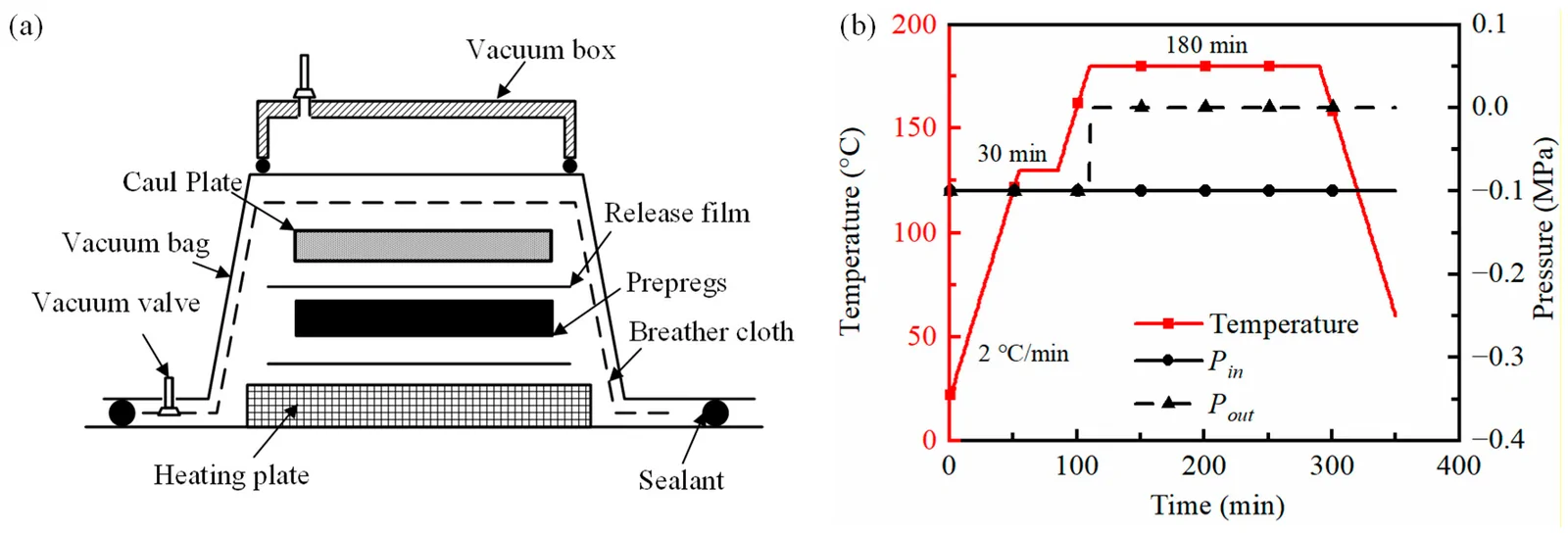
Equipment and curing cycle of DVB process: (**a**) equipment; (**b**) curing cycle.

The equipment and curing cycle used in this paper are shown in Figure 1. The heating uniformity of the heating plate is ± 2 °C, and the heating rate can be adjusted. The outer bag of the DVB process was replaced by a vacuum box, and a sealing strip was used to form a vacuum chamber with the vacuum bag to pull up the vacuum bag. The vacuum box and vacuum bag were connected to two vacuum pumps respectively; this allowed the vacuum level of the two chambers to be adjusted independently. In the curing cycle of the DVB process, the heating rate throughout the entire curing cycle was 2 °C/min. Based on previous explorations [31,32], the operation of raising the temperature from room temperature to 130 °C and maintaining it for 30 min was adopted, which can effectively remove gases inside prepregs. Then the temperature rose to 180 °C and was maintained for 180 min for the material to complete curing. Finally, the laminate was removed after it was cooled to 60 °C. The pressure of the vacuum bag and vacuum box is represented by *P_in_* and *P_out_*. Before the end of the first insulation treatment, the vacuum bag and vacuum box were completely evacuated. Afterwards, the vacuum box chamber was connected to the atmosphere, and the vacuum bag was still subject to vacuum treatment. In order to compare the void content and mechanical properties of composites under different processes, the SVB process was set as the control group in this study. Unlike the DVB process, the SVB process did not undergo the first insulation treatment and directly heated the material up to 180 °C with a 2 °C/min heating rate. Throughout the entire curing cycle, only vacuum bags were used to compact the material.

### 2.3. Physical Property Test

Compared with other methods, the void morphology can be observed cleanly by microscopy [33,34,35]. In this paper, samples prepared by different processes were cut from laminates. After grinding and polishing, the cross-section images of samples were photographed with a super depth-of-field microscope VHX-6000 (Keyence Corporation, Osaka, Japan). The image analysis software ImageJ-1.53t was used to measure and calculate the void content. According to the ASTM D792-20 and ASTM D3171-22 testing standards [36,37], the Archimedes drainage method is used to test the sample density, and the burning method is used to test the sample fiber volume fraction. Five samples were used for each test.

### 2.4. Mechanical Properties Test

In order to compare the mechanical properties of materials under different processes, the three-point bending test and short-beam-shear test were conducted. The three-point bending test was conducted according to the ASTM D7264-21 testing standard at a loading rate of 1 mm/min [38]. The short-beam-shear test was used to measure the material ILSS and was conducted according to the ASTM D2344-22 testing standard, with a loading rate of 1 mm/min [39]. Five samples were used for each test.

## 3. Results and Discussion

### 3.1. Different Stacking Angles

**Figure 2:**
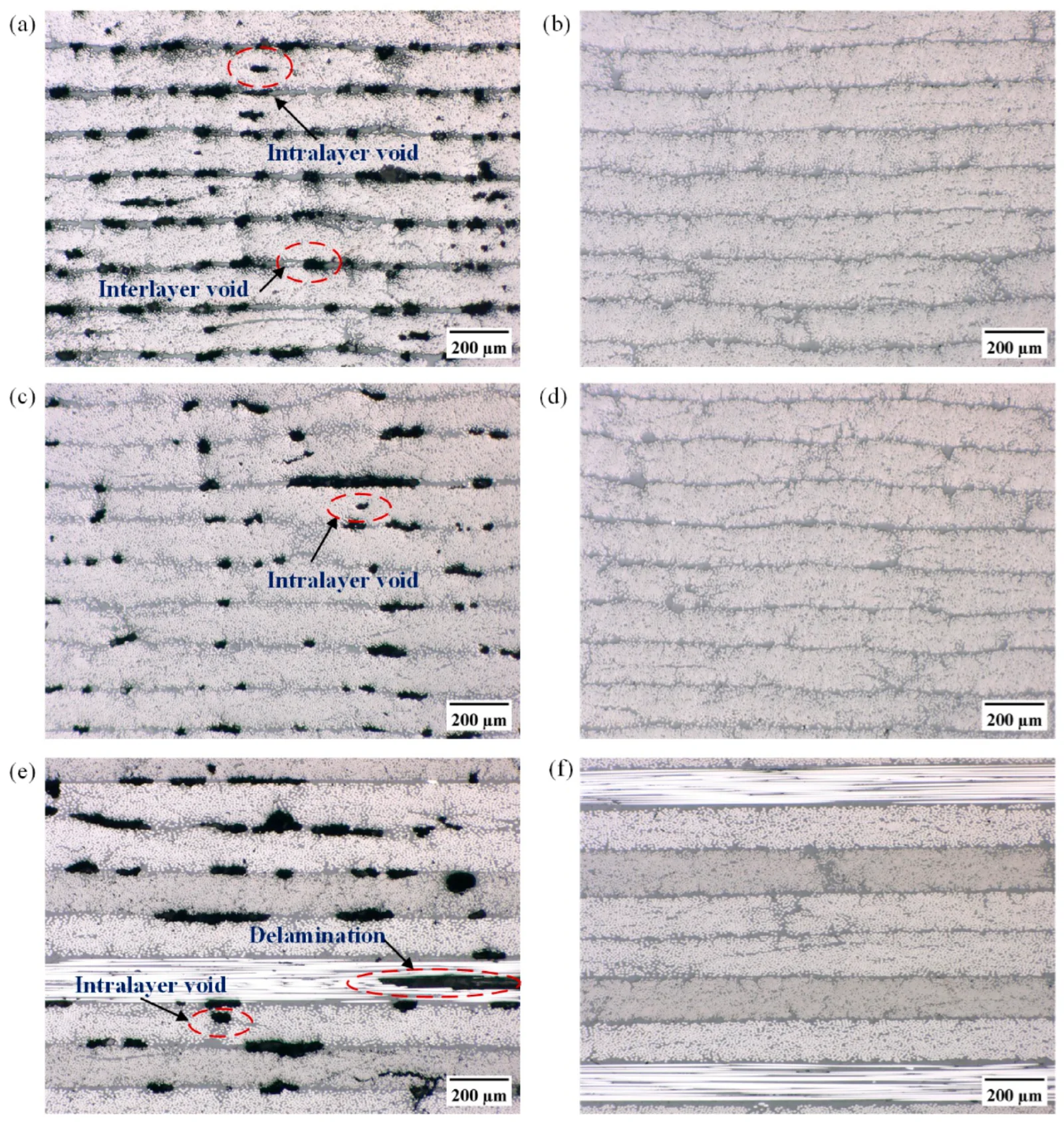
Cross-section microscope images of composites with different stacking angles: (**a**) SVB-[0]_16_; (**b**) DVB-[0]_16_; (**c**) SVB-[90]_16_; (**d**) DVB-[90]_16_; (**e**) SVB-[0/+45/90/−45]_4S_; (**f**) DVB-[0/+45/90/−45]_4S_.

Figure 2 shows microscopic images of composite cross-sections at different stacking angles. It can be seen that the materials under the SVB process with different stacking angles all contain a large number of internal voids. The voids were mainly concentrated in the resin area between layers, and the aggregation and fusion of voids cause delamination. Meanwhile, there are a small number of voids inside layers. From Table 2, the void content of the [0]_16_ laying material is 8.27%, the void content of the [90]_16_ laying material is 4.59%, and the void content of the [0/+45/90/−45]_4S_ laying material is 5.21%. On the contrary, the materials with different stacking angles generally do not contain voids from the DVB process, and the thin-layer resin was tightly attached between the layers. The void contents of materials with three different layer angles are 0.01%, 0.01% and 0.04%, respectively, which are all lower than that of the SVB process and less than 1%.

**Figure 3:**
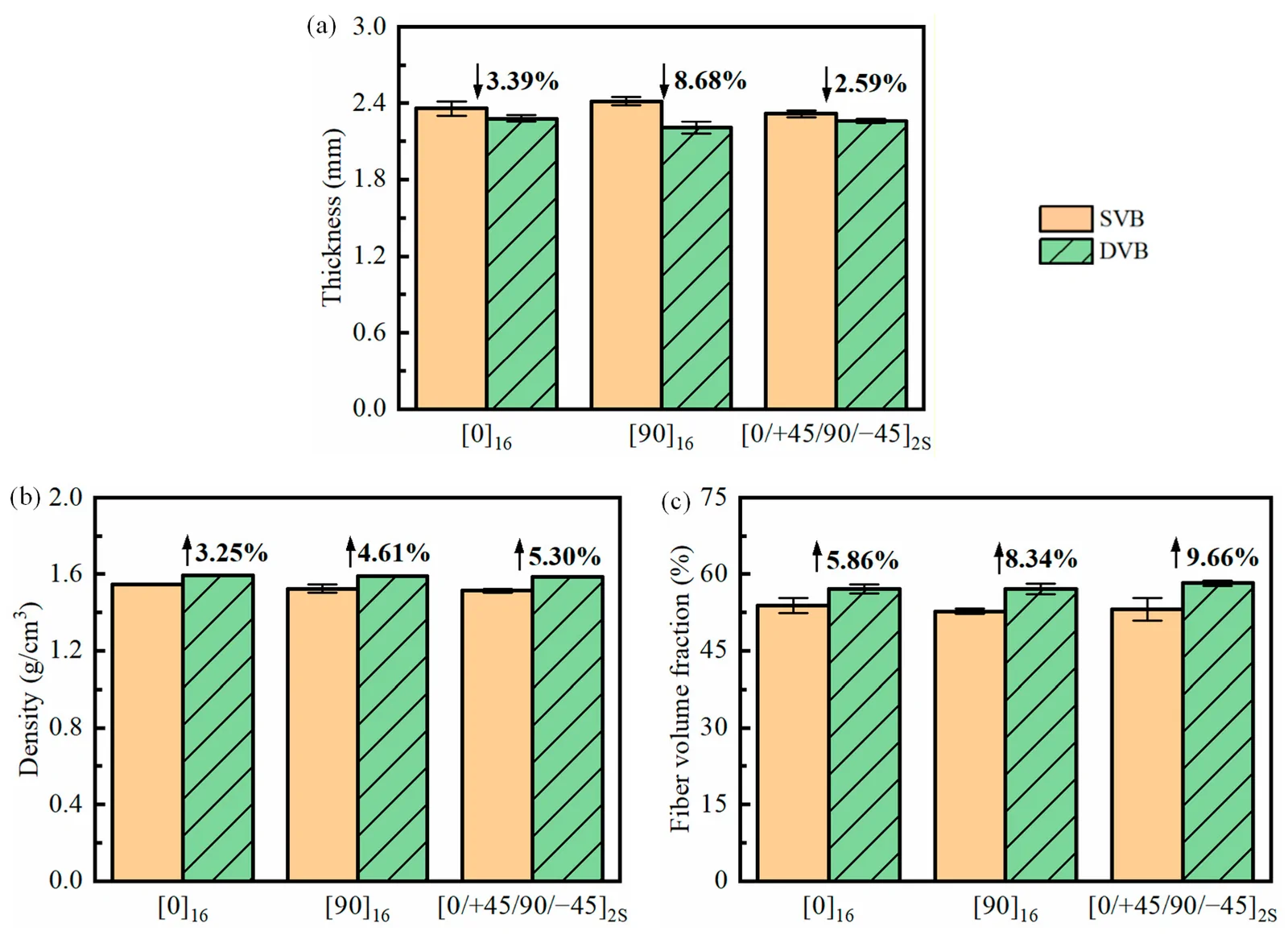
Physical properties of composites with different stacking angles: (**a**) thickness, (**b**) density, and (**c**) fiber volume fraction.

From Figure 3, it can be seen that the physical properties of composites with different stacking angles are different. Under the SVB process, the composite thicknesses are 2.36 mm, 2.42 mm and 2.32 mm, respectively; the densities are 1.54 g/cm^3^, 1.52 g/cm^3^, and 1.51 g/cm^3^, respectively; and the fiber volume fractions are 53.90%, 52.73% and 53.09%, respectively. Under the DVB process, the laminate thicknesses are 2.28 mm, 2.21 mm and 2.26 mm, respectively; the densities are all 1.59 g/cm^3^; and the fiber volume fractions are 57.06%, 57.13% and 58.22%, respectively. For composite materials with different stacking angles, it can be found that the DVB process reduces the composite void content and tightly compacts the laminate because the air trapped between layers is effectively extracted.

**Figure 4:**
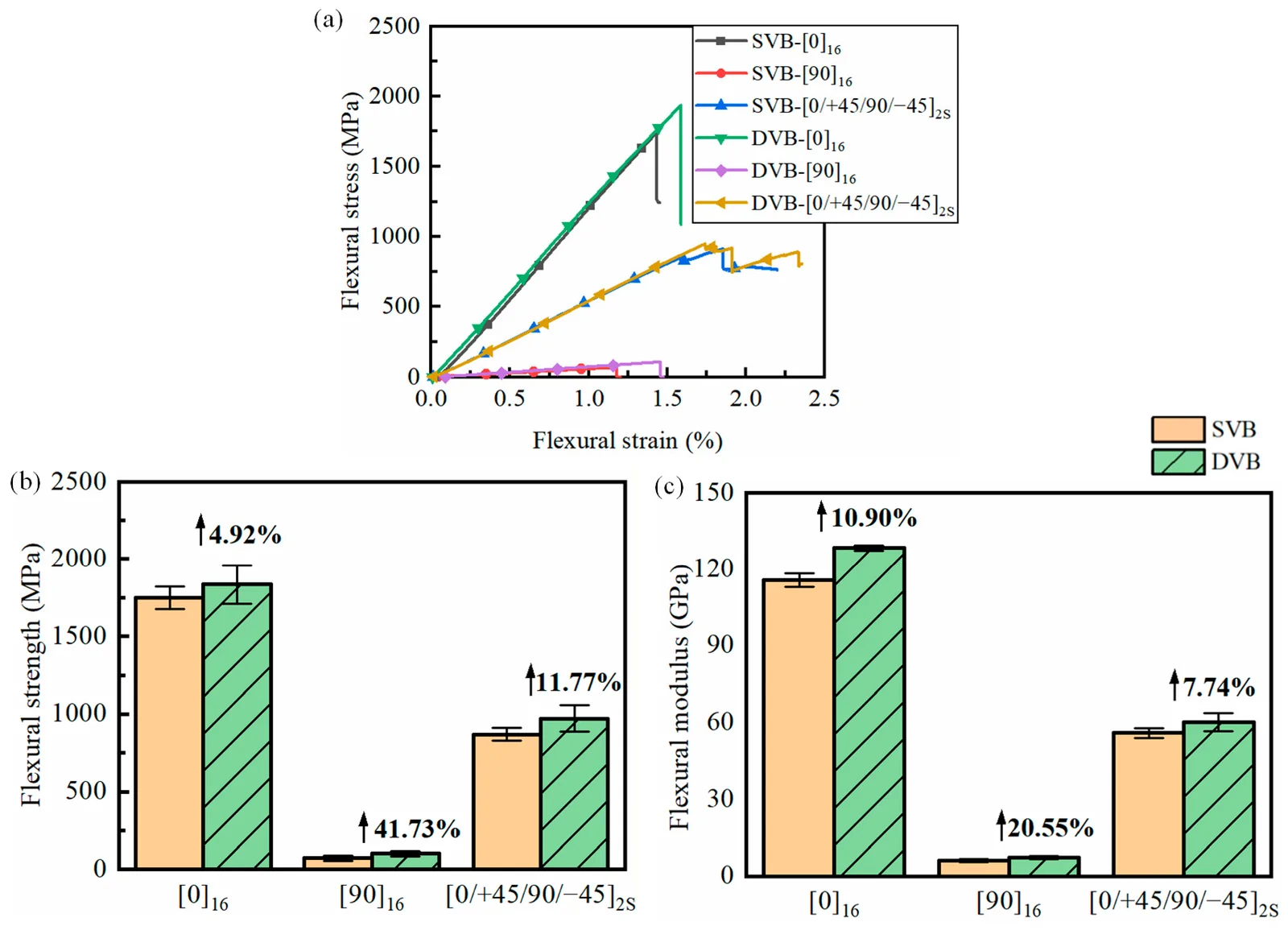
Flexural properties of composites with different stacking angles: (**a**) stress–strain curve; (**b**) flexural strength; (**c**) flexural modulus.

Figure 4 shows the mechanical properties of specimens with different stacking angles under bending loads. All specimens failed after reaching the ultimate load, resulting in specimen failure. The results show that the flexural strengths of the composites with three different stacking angles under the SVB process are 1749 MPa, 72.10 MPa, and 869.8 MPa, respectively, and the flexural moduli are 115.7 GPa, 6.13 GPa, and 55.91 GPa respectively. The flexural strengths under the DVB process are 1835 MPa, 102.2 MPa, and 972.1 MPa, respectively, and the flexural moduli are 128.3 GPa, 7.39 GPa, and 60.24 GPa. It can be concluded that the flexural performance of composite materials prepared by the DVB process with different layer angles is superior to that of the SVB process. It can be seen that the load-bearing capacity of the [0]_16_ laying material is the strongest, the [0/+45/90/−45]_4S_ laying material is second, and the [90]_16_ laying material is the weakest. This is because the flexural resistance of the 0° layer is the strongest, while the flexural resistance of the 90° layer is insufficient. In the [90]_16_ laying material, the 90° layer relies solely on resin to withstand flexural loads, and the material fractures and fails due to shear forces under small loads, resulting in complete fracture of the entire specimen.

**Figure 5:**
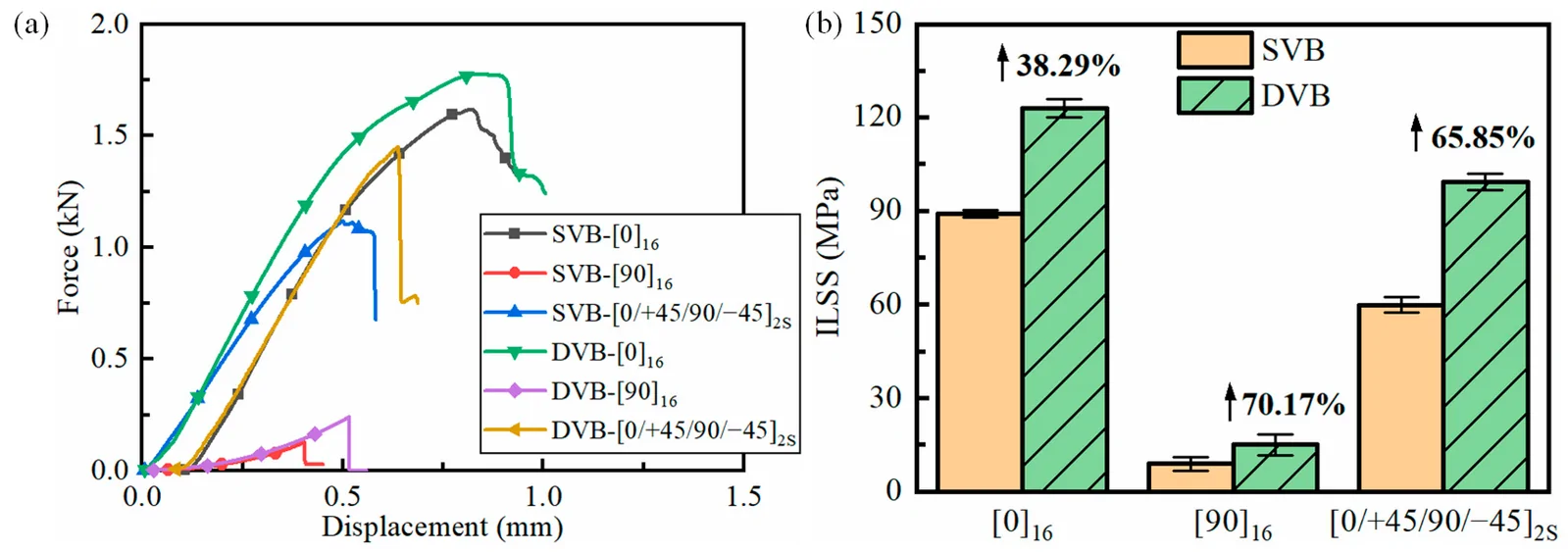
ILSS test results of composites with different layer angles: (**a**) force–displacement curve; (**b**) ILSS.

Figure 5 shows the mechanical properties of specimens in the ILSS test. All specimens experienced a rapid decrease in force after reaching the ultimate load. The ILSS of the three stacking angles composites under the DVB process are 123.0 MPa, 15.06 MPa, and 99.23 MPa, respectively, while the ILSS of the SVB process are 88.96 MPa, 8.85 MPa, and 59.83 MPa, which is an increase of 38.29%, 70.17%, and 65.85% respectively. Voids can cause stress and strain concentration, and cracks are more likely to initiate and expand around voids, thereby reducing the mechanical properties of composites. Compared with the flexural strength, the ILSS of composite materials with different stacking angles exhibits strong void sensitivity.

### 3.2. Different Stacking Thicknesses

**Figure 6:**
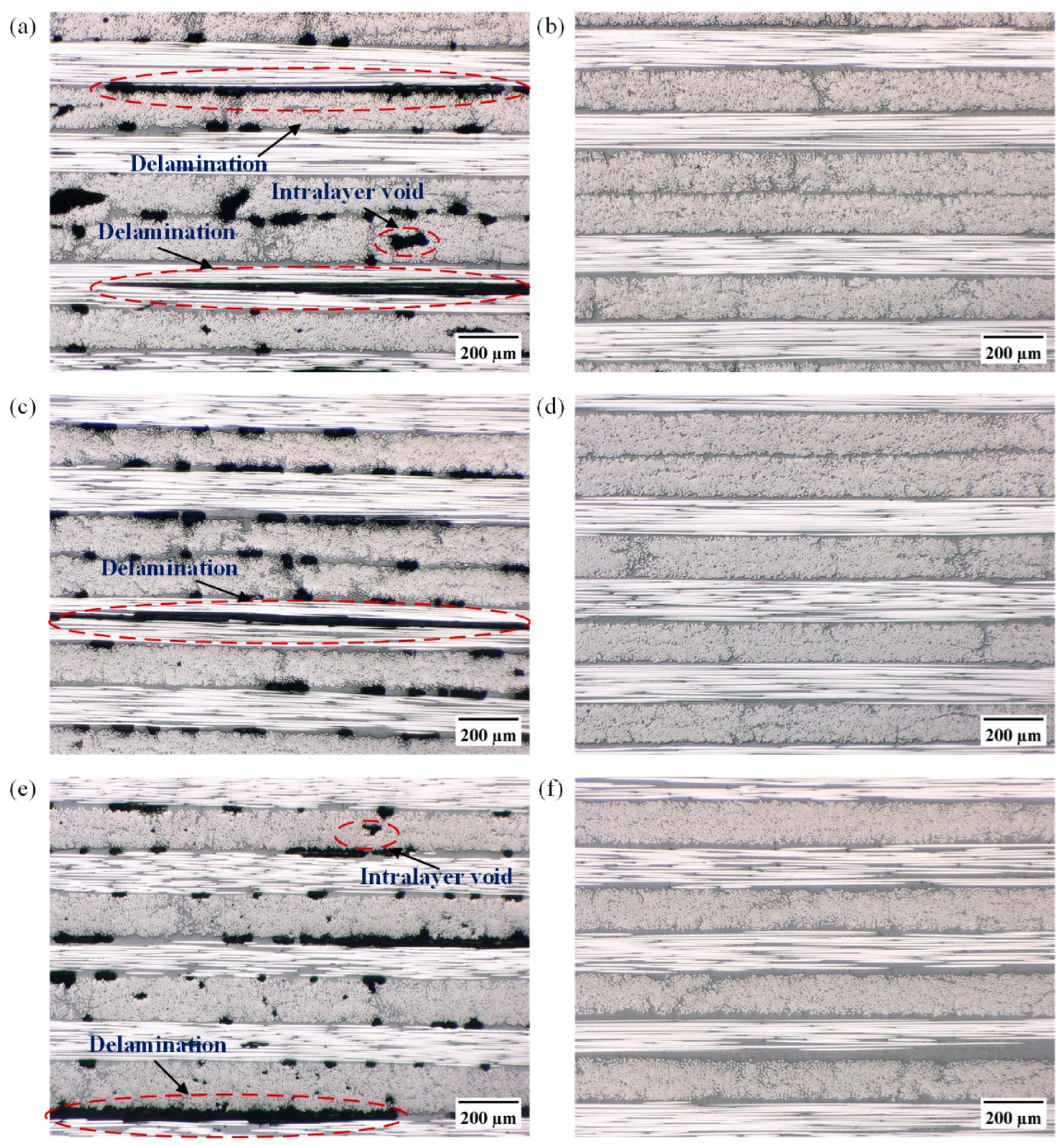
Cross-section microscope images of composites with different stacking thicknesses: (**a**) SVB-[0/90]_3S_; (**b**) DVB-[0/90]_3S_; (**c**) SVB-[0/90]_5S_; (**d**) DVB-[0/90]_5S_; (**e**) SVB-[0/90]_7S_; (**f**) DVB-[0/90]_7S_.

As shown in Figure 6, materials with different laying thicknesses contain a large number of voids with the SVB process. Delamination occurs within and between layers, and there are a few voids inside a layer, which indicates that the wetness of the fiber and resin became worse. Under the SVB process, the void contents of [0/90]_3S_, [0/90]_5S_, and [0/90]_7S_ laying materials are 8.26%, 8.97%, and 6.84%, respectively, as shown in Table 3. The void contents decreased significantly to 0.04%, 0.01%, and 0.02%, respectively with the DVB process. From the above results, it can be seen that the DVB process can still effectively remove the air from the material and reduce the void content with different thicknesses.

**Figure 7:**
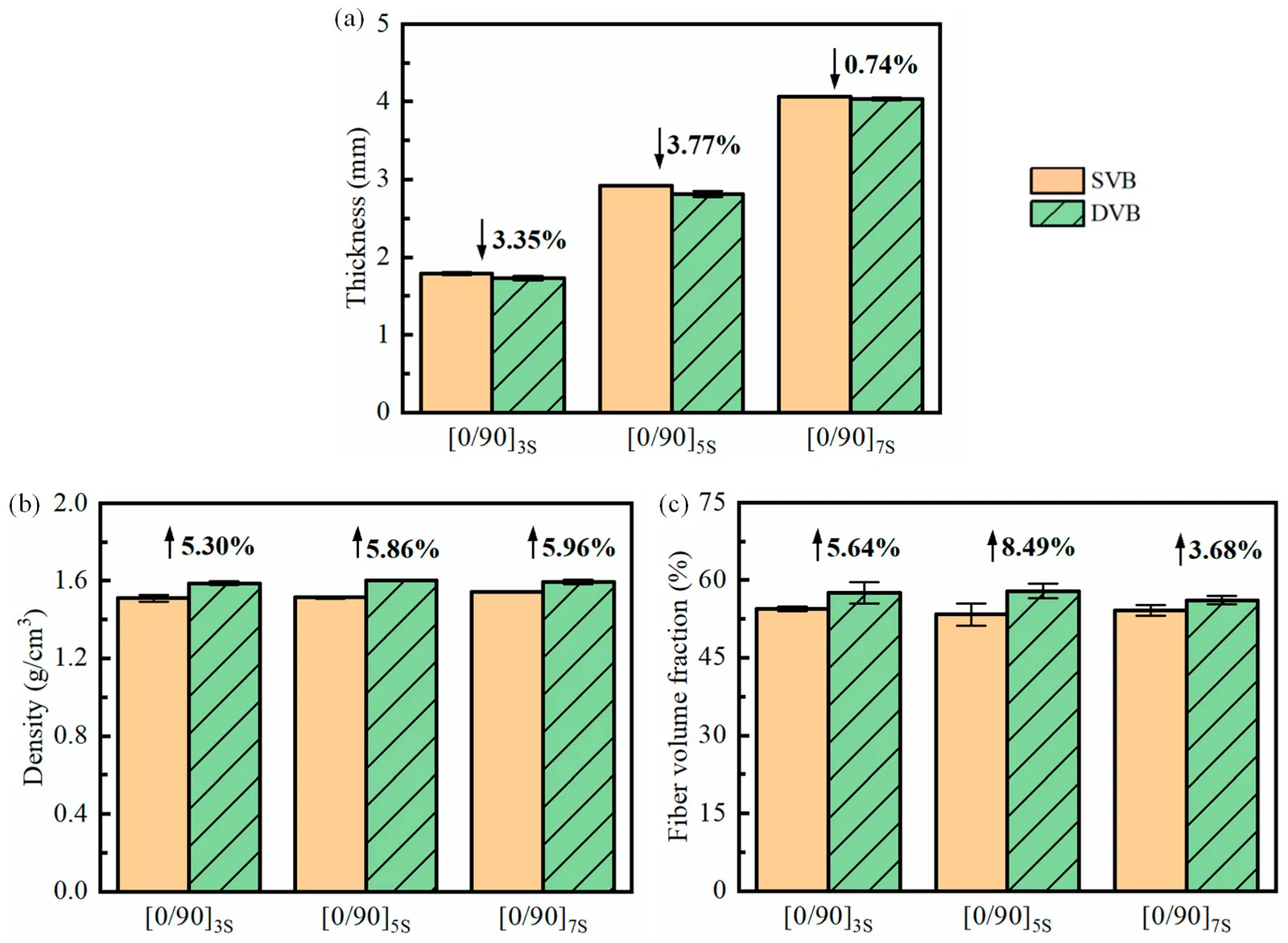
Physical properties of composites with different stacking thicknesses: (**a**) thickness; (**b**) density; (**c**) fiber volume fraction.

The physical properties of composite materials with different stacking thicknesses are shown in Figure 7. Under the SVB process, the composite thicknesses with three different laying thicknesses are 1.79 mm, 2.92 mm, and 4.06 mm; the densities are 1.51 g/cm^3^, 1.51 g/cm^3^, and 1.54 g/cm^3^; and the fiber volume fractions are 54.43%, 53.37%, and 54.13% respectively. Compared with the SVB process, the composite thickness becomes thinner, and the densities and the fiber volume fractions increase with the DVB process. It can be inferred that the laminates prepared by the DVB process become denser with different layer thicknesses. The low pressure of the SVB process results in the inability to compact materials.

**Figure 8:**
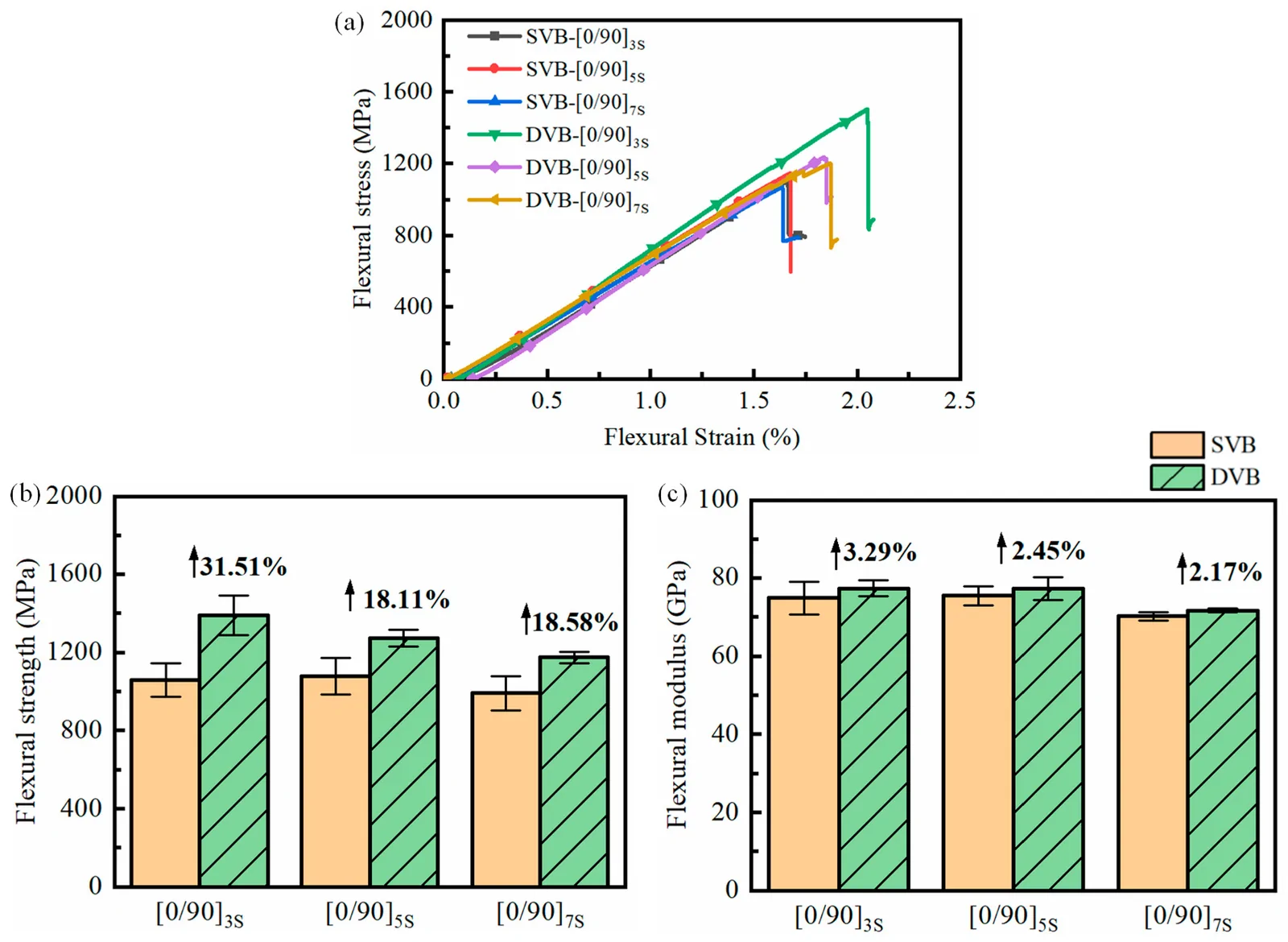
Flexural properties of composites with different stacking thicknesses: (**a**) stress–strain curve; (**b**) flexural strength; (**c**) flexural modulus.

Figure 8 shows the mechanical properties of composite specimens with different stacking thicknesses. The results show that the flexural strengths of the composites are 1058 MPa, 1077 MPa, and 990.8 MPa for the SVB process. Compared with this, the flexural strengths of the DVB process increased by 31.51%, 18.11%, and 18.58% respectively. The increase in flexural modulus is small because it depends on the fiber. It is clear that the void can reduce the flexural bearing capacity of composite materials with different stacking thicknesses.

**Figure 9:**
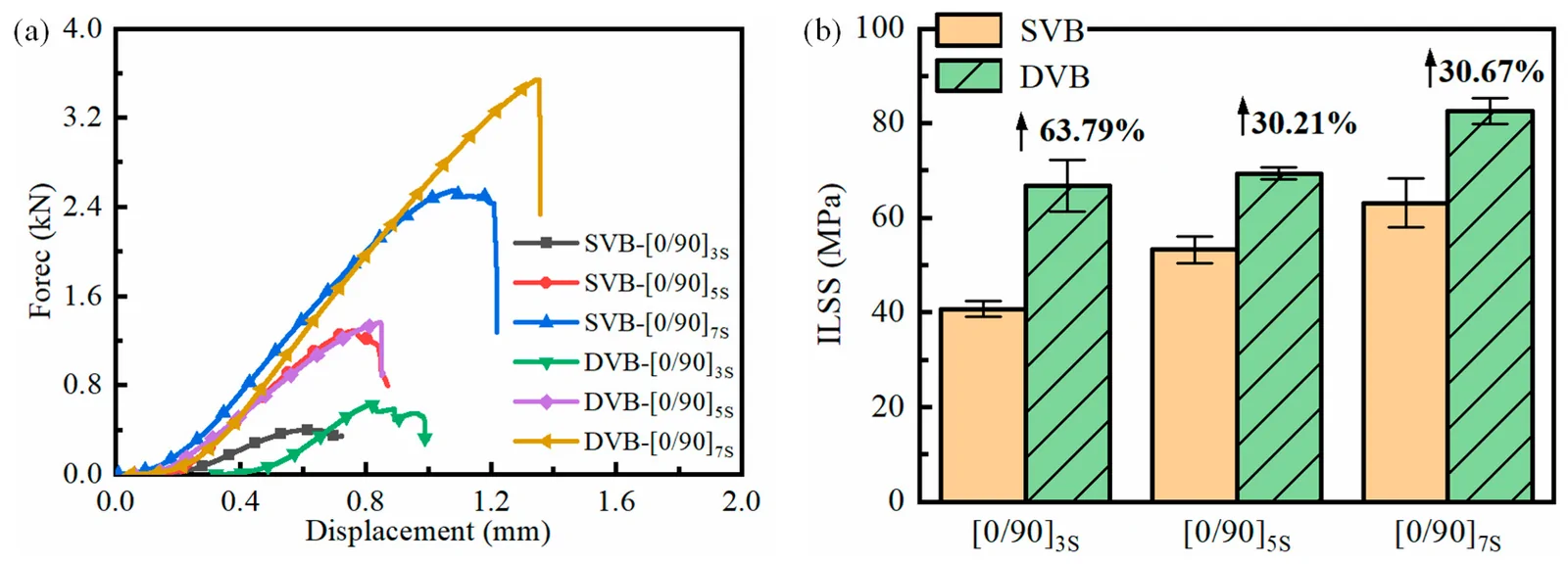
ILSS test results of composites with different stacking thicknesses: (**a**) force–displacement curve; (**b**) ILSS.

Figure 9 shows the mechanical properties of ILSS tests on composite specimens with different stacking thicknesses. As the thickness increases, the ultimate load that can be withstood increases. The ILSS of the composite materials for the DVB process are 66.71 MPa, 69.31 MPa, 82.49 MPa, and 40.73 MPa, 53.23 MPa, 63.14 MPa for the SVB process, with increases of 63.79%, 30.21%, and 30.67% respectively. From the test results, it can be seen that the ILSS of composite materials with different stacking thicknesses prepared by the DVB process is superior to that of the SVB process.

### 3.3. Plain Weave Material

**Figure 10:**
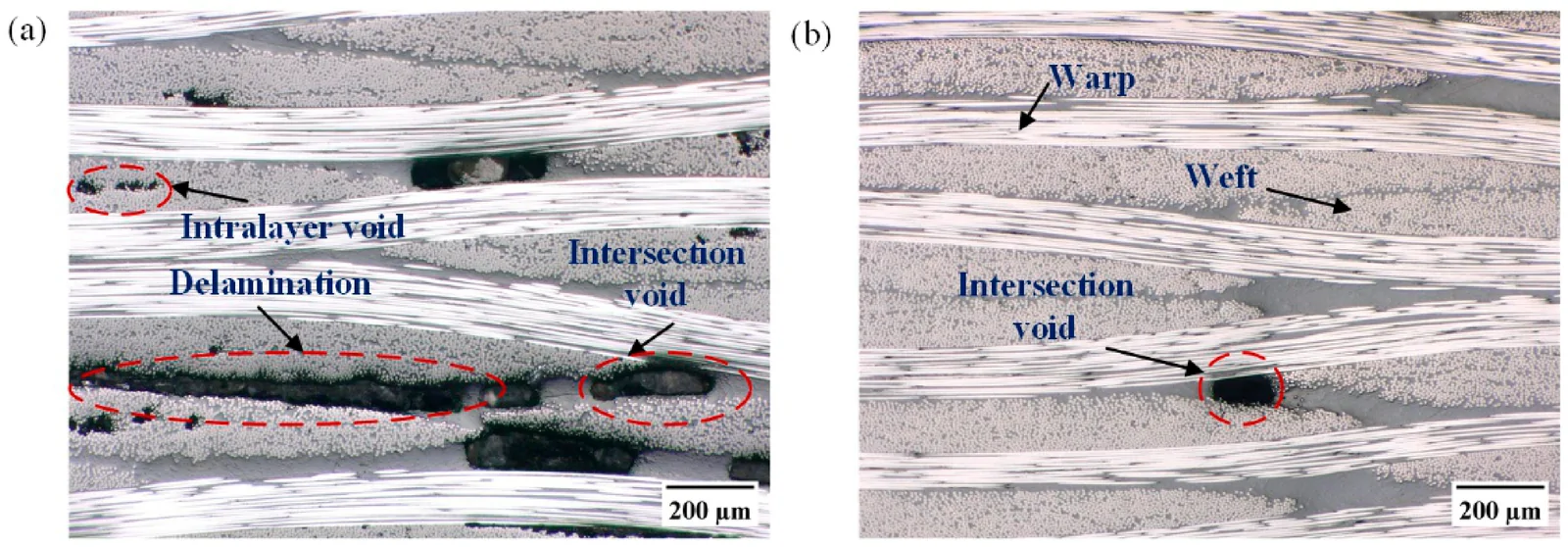
Cross-section microscope images of plain weave composites: (**a**) SVB process; (**b**) DVB process.

Figure 10 shows the cross-section microscopic images of plain weave composites under different processes. Unlike unidirectional prepregs, the fiber reinforcement contains warp and weft fiber bundles; thus, the intersection area is not easily infiltrated by resin, leading to air accumulation and void formation. In Figure 10, it can be observed that a large number of voids are concentrated at the intersection area for the SVB process. The void content reaches 9.12% in Table 4: the large voids between layers cause delamination, and there are a small number of voids inside layers. The number of voids is significantly reduced under the DVB process: only a small number of voids are left at the intersections, with a void content of 0.23%, which still meets the requirement of a void content less than 1% for aerospace components. Moreover, the material thickness decreases from 2.78 mm to 2.66 mm compared with the SVB process; the density increases from 1.48 g/cm^3^ to 1.52 g/cm^3^; and the fiber volume fraction increases from 50.19% to 51.47%, proving that the material’s tightness increases.

**Figure 11:**
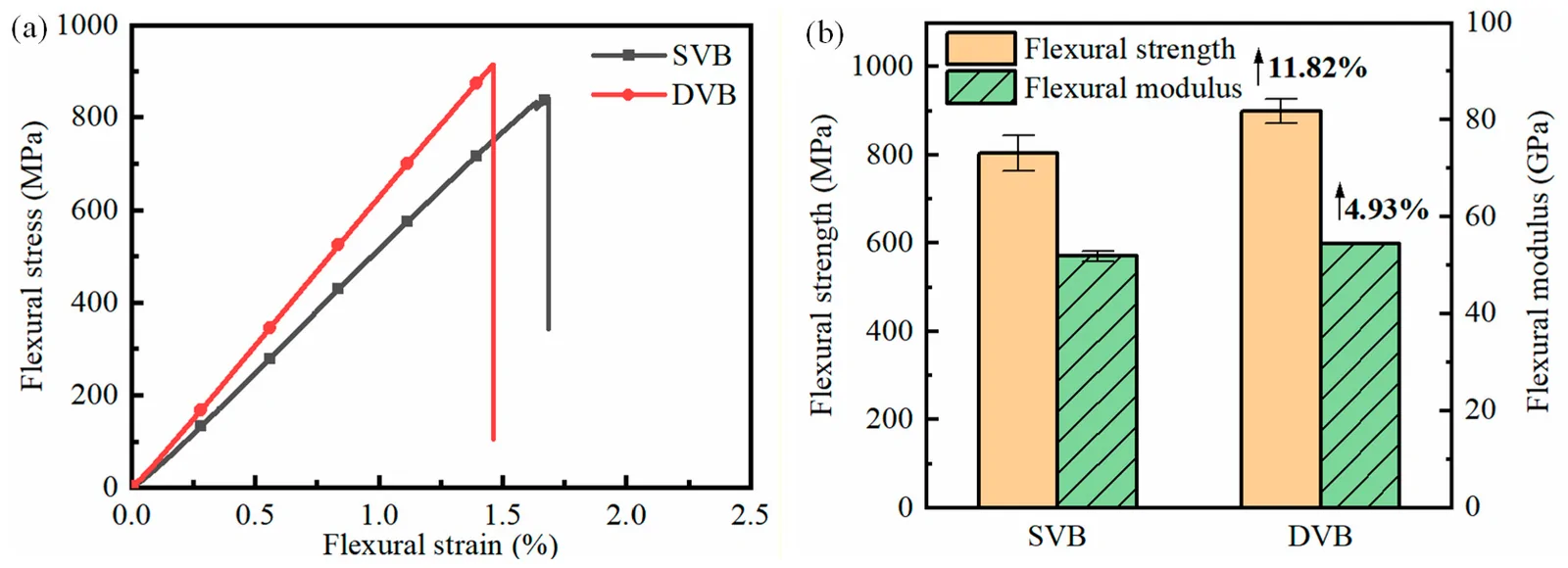
Flexural performance of plain weave composites: (**a**) stress–strain curve; (**b**) flexural strength and flexural modulus.

Figure 11 shows the flexural mechanical properties of plain weave composite specimens; all specimens completely fracture under ultimate load. The flexural strength of the DVB process is 899.0 MPa; the flexural strength of the SVB process is 804.2 MPa, increasing by 11.82%. The increase in flexural modulus is only 4.93%. Unlike unidirectional composite materials, plain weave composite samples completely fracture under flexural load. This is because the material adopts a [(0,90)]_10_-layer structure; the warp fibers in each layer have good bending resistance, thereby increasing the composite stiffness. In addition, the fiber bundle intersection causes warp and weft fibers to bend, resulting in stress concentration at the intersection area; this makes stress distribution more complex. The presence of voids can exacerbate the degree of stress concentration: cracks can be propagated between voids in samples and fiber bundles tend to break around voids first. Therefore, the flexural strength of the SVB process is reduced.

**Figure 12:**
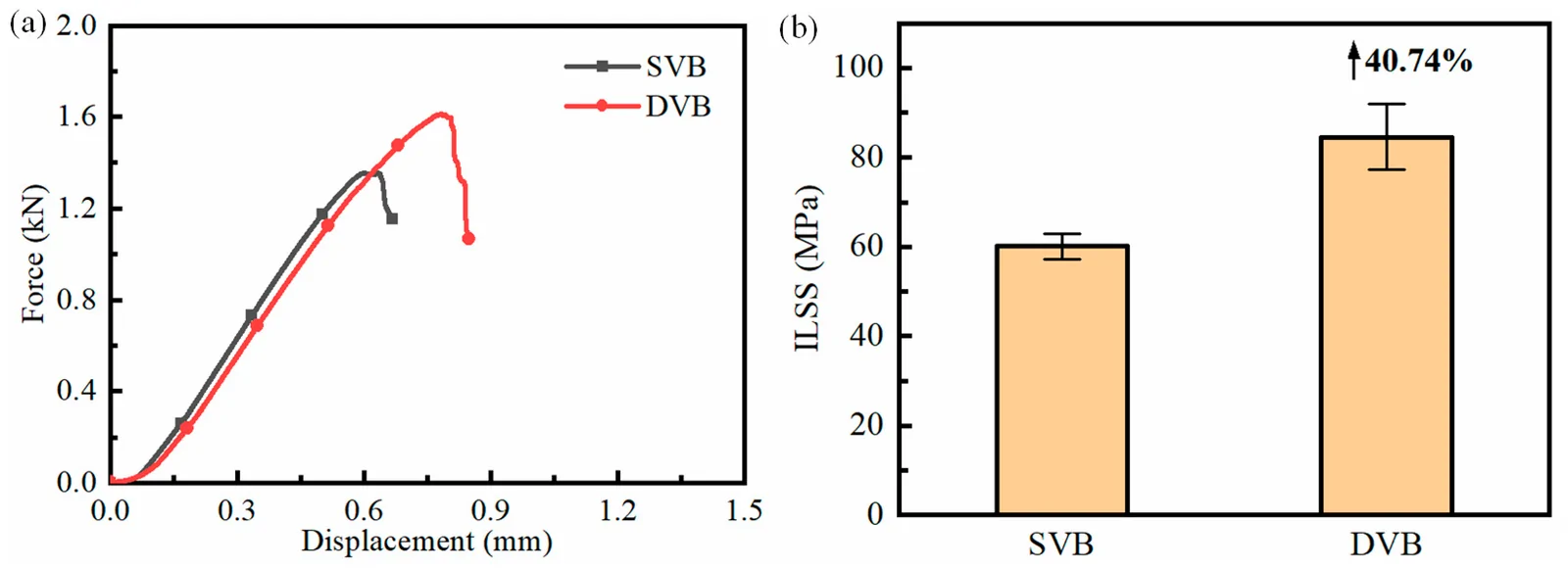
ILSS test results of plain weave composites: (**a**) force–displacement curve; (**b**) ILSS.

Figure 12 shows the force–displacement curve of the ILSS test of plain weave composites. The material-bearing capacity of the SVB process is smaller than that of the DVB process. It can be seen that the ILSS of the DVB process is 84.60 MPa, increasing by 40.74% compared with the SVB process.

## 4. Conclusions

In order to investigate the effect of the DVB process on composite materials with different stacking sequences, laminates with different stacking angles, stacking thicknesses, and plain weave composites were manufactured by the DVB process, and the SVB process was set as the control group. The void morphology of different laminates was observed using a microscope; the thickness, density and fiber volume fraction of laminates were measured; and the flexural performance and ILSS of laminates were evaluated.

The study results show that the void contents of all laminates are lower than 1% with the DVB process, which meets the application requirements of aerospace composite components. In the SVB process, there are a lot of voids inside the composites; the thickness of laminates increases; and the density and fiber volume fraction decrease, compared with the DVB process. For plain weave composites, it is difficult for resin to infiltrate the intersection of warp and weft yarns, which can easily form voids; there are still a few voids left with the DVB process, but it is much lower than the SVB process. Mechanical tests show that the flexural strength and ILSS of the SVB process are lower than the DVB process due to the existence of internal voids, and ILSS shows stronger void sensitivity. Voids can reduce the mechanical properties of composites.

The results of this paper broaden the application range of the DVB process and confirm that the DVB process can effectively reduce the void content of composites with different laying modes, maintaining their mechanical properties.

## Figures and Tables

**Table 1:** Stacking sequences of prepregs.

Stacking Sequences	Laying Sequences	Layer Quantity
Stacking angles	[0]_16_	16
	[90]_16_	16
	[0/+45/90/−45]_4S_	16
Stacking thicknesses	[0/90]_3S_	12
	[0/90]_5S_	20
	[0/90]_7S_	28
Plain weave	[(0,90)]_10_	10

**Table 2:** Void content of composites with different stacking angles.

Stacking Angles	Void Content (%)
SVB-[0]_16_	8.27 ± 1.26
DVB-[0]_16_	0.01 ± 0.01
SVB-[90]_16_	4.59 ± 0.94
DVB-[90]_16_	0.01 ± 0.01
SVB-[0/+45/90/−45]_4S_	5.21 ± 1.40
DVB-[0/+45/90/−45]_4S_	0.04 ± 0.02

**Table 3:** Void content of composites with different laying thicknesses.

Stacking Angles	Void Content (%)
SVB-[0/90]_3S_	8.26 ± 1.30
DVB-[0/90]_3S_	0.04 ± 0.04
SVB-[0/90]_5S_	8.97 ± 1.73
DVB-[0/90]_5S_	0.01 ± 0.01
SVB- [0/90]_7S_	6.84 ± 1.89
DVB-[0/90]_7S_	0.02 ± 0.05

**Table 4:** Physical properties of plain weave composites.

Physical Properties	SVB	DVB	Percentage (%)
Void content (%)	9.12 ± 2.28	0.23 ± 0.38	−97.48
Thickness (mm)	2.78 ± 0.02	2.66 ± 0.01	−4.32
Density (g/cm^3^)	1.48 ± 0.02	1.52 ± 0.01	+2.70
Fiber volume fraction (%)	50.19 ± 2.17	51.47 ± 1.15	+2.55

## Data Availability

The raw data supporting the conclusions of this article will be made available by the authors on request.

## Abbreviations

The following abbreviations were used within the manuscript:

DVB	Double vacuum bag
SVB	Single vacuum bag
ILSS	Interlayer shear strength
OoA	Out-of-autoclave
ASTM	American Society for Testing and Materials
